# Antioxidant and Hepatoprotective Activity of *Veronica ciliata* Fisch. Extracts Against Carbon Tetrachloride-Induced Liver Injury in Mice

**DOI:** 10.3390/molecules19067223

**Published:** 2014-06-02

**Authors:** Li Yin, Lei Wei, Rao Fu, Lisheng Ding, Yiran Guo, Lin Tang, Fang Chen

**Affiliations:** 1 College of Life Sciences, Sichuan University, Chengdu 610064, China; E-Mails: yinli0130@gmail.com (L.Y.); yhweilei@163.com (L.W.); furao926@gmail.com (R.F.); ran@scu.edu.cn (Y.G.); 2 Sichuan Key Laboratory of Resource Biology and Biopharmaceutical Engineering, Key Laboratory of Bio-resources and Eco-environment, Ministry of Education, Chengdu 610064, China; 3 Key Laboratory of Mountain Ecological Restoration and Bioresource Utilization, Chengdu Institute of Biology, Chinese Academy of Sciences, Chengdu 610041, China; E-Mail: dinglisheng613@gmail.com

**Keywords:** *Veronica ciliata* Fisch, antioxidant activity, hepatoprotective effect, carbon tetrachloride, histological examination

## Abstract

*Veronica ciliata* Fisch. has been traditionally used in Traditional Chinese Medicine prescriptions due to its curative effects for hepatitis, cholecystitis, rheumatism, and urticaria. The present study was focused on investigating the role of ethyl acetate and aqueous extracts of *Veronica ciliata* Fisch. Furthermore, *in vitro* antioxidant activity (scavenging of DPPH, ABTS, superoxide, and nitrite radicals; reducing power; β-carotene bleaching) and the hepatoprotective effect of the ethyl acetate extract by means of CCl_4_-induced oxidative stress in mice were investigated. The ethyl acetate extract of *Veronica ciliata* Fisch. displayed more noteworthy *in vitro* antioxidant activities than the aqueous extract. Moreover, it significantly prevented the increase in serum T-AOC, ALT, AST and ALP level in acute liver damage induced by CCl_4_, decreased the extent of MDA formation in liver and elevated the activities of SOD and GSH in liver. This activity was found to be comparable to that of bifendate. Histopathological observation of the liver was also performed to further support the evidence from the biochemical analysis. The results indicated that strong antioxidant activities and a significant protective effect against acute hepatotoxicity induced by CCl_4_ of *Veronica ciliata* Fisch. were concentrated in the ethyl acetate extract. The results suggested that this activity may be due to free radical-scavenging and antioxidant properties.

## 1. Introduction

*Veronica ciliata* Fisch. is an annual herb belonging to the Scrophulariaceae which mainly occurs in the northwest territories, northern Sichuan, and the Tibetan autonomous region (China). Traditionally, *V. ciliata* Fisch. has been commonly used in the Tibetan medicine prescription due to its curative effect for hepatitis, cholecystitis, rheumatism, and urticaria [1]. Five iridoid glycosides and three benzoic acid derivatives have been isolated from *V.* *ciliata* Fisch. [2], but very little, if any, research has been done on the antioxidant activities of *V.* *ciliata* Fisch. extracts (VCFE), especially examining their hepatoprotective effect on CCl_4_-induced acute liver damage in mice.

The liver is a vital organ and has many important functions and liver diseases have become one of the major causes of morbidity and mortality in man and animals all over globe and hepatotoxicity due to drugs appears to be the most common contributing factor [3]. Carbon tetrachloride (CCl_4_) is a widely used hepatotoxic agent in rodents and trichloromethyl radical (CCl_3_)-induced toxicity in rat liver closely resembles human cirrhosis [4]. Liver-protective plants include a variety of chemical constituents like phenols, coumarins, lignans, essential oil, monoterpenes, carotinoids, glycosides, flavanoids, organic acids, lipids, alkaloids and xanthenes [5]. Lots of studies indicate that natural products from edible and medicinal plants exhibit strong antioxidant activity that could act against CCl_4_-induced liver damage [6,7,8,9,10], because they contain lots of free radical scavengers.

The present study was focused on investigating the role of ethyl acetate and aqueous extracts (AE) from *V.* *ciliata* Fisch. against CCl_4_-induced hepatic injury. The antioxidant activity of AE and EAE was evaluated *in vitro*. To evaluate the hepatoprotective effect of VCFE in the *in vivo* study, the serum levels of different marker enzymes related to hepatic integrity such as total antioxidative capacity (T-AOC), alanine amino transferase (ALT), aspartate amino transferase (AST), and alkaline phosphatase (ALP) were determined. To evaluate the effect of VCFE on the CCl_4_-induced cellular levels of total protein (TP), malondialdehyde (MDA), superoxide dismutase (SOD) and glutathione (GSH) in the liver were also determined. In addition, histological studies were done to prove the effectiveness of VCFE in a preventive and curative role against CCl_4_-induced toxicity. The results indicated that ethyl acetate extracts (EAE) from *V.* *ciliata* Fisch. have stronger antioxidant activities than aqueous extracts and show significant protective effects against acute hepatotoxicity induced by CCl_4_, and this is supported by the evaluation of liver histopathology in mice. The results suggested that the possible mechanism of this activity may be due to free radical-scavenging and antioxidant activity.

## 2. Results and Discussion

### 2.1. Total Phenolic and Flavonoid Content

Phenolic and flavonoid compounds of plant extracts are recognized as active substances responsible for the antioxidant activity due to their free radical scavenging effects. The total phenolic contents of AE and EAE were 112.19 ± 23.61 and 264.29 ± 13.64 mg/g gallic acid equivalent, and the total flavonoid content was 78.11 ± 9.53 and 157.29 ± 16.31 mg/g rutin equivalent, respectively.

### 2.2. In Vitro Antioxidant Activity Assays

**Figure 1:**
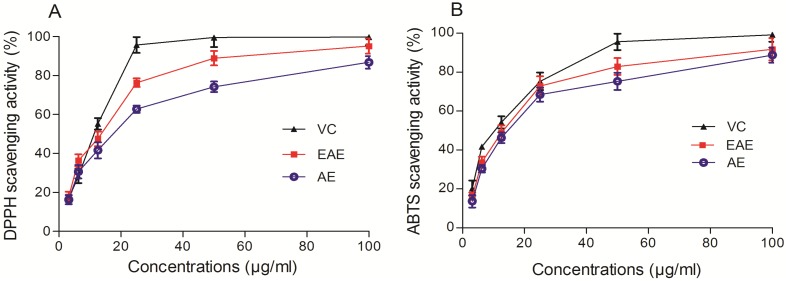
*In vitro* free radical scavenging of ethyl acetate and aqueous extracts from *V. ciliata* Fisch. (**A**) DPPH scavenging activity of various concentrations of VCFE and reference VC. (**B**) ABTS radical scavenging activity of VCFE and VC. Data are presented as means ± SD (*n* = 3). VCFE, *V.* *ciliata* Fisch. Extracts; VC, Vitamin C; EAE, ethyl acetate extracts of *V.* *ciliata* Fisch.; AE, aqueous extracts of *V.* *ciliata* Fisch.

As shown in Figure 1A, VCFE displayed concentration-dependently DPPH scavenging effects at the tested concentrations of 3.125–100 μg/mL. In the DPPH scavenging assay, the IC_50_ values (the concentration required to scavenge 50% of radical) of EAE, AE and VC were 11.08 ± 0.022, 20.03 ± 0.035 and 9.769 ± 0.020 μg/mL. In the ABTS assay (Figure 1B), VCFE was found to be an efficient ABTS radical scavenger and the activity was dose dependent (3.125–100 μg/mL). The IC_50_ values of EAE, AE and VC were 13.25 ± 0.017, 19.64 ± 0.053 and 11.62 ± 0.032 μg/mL, respectively. The result suggests that VCFE shows significant scavenging activity and it is comparable with the standard VC. 

**Figure 2:**
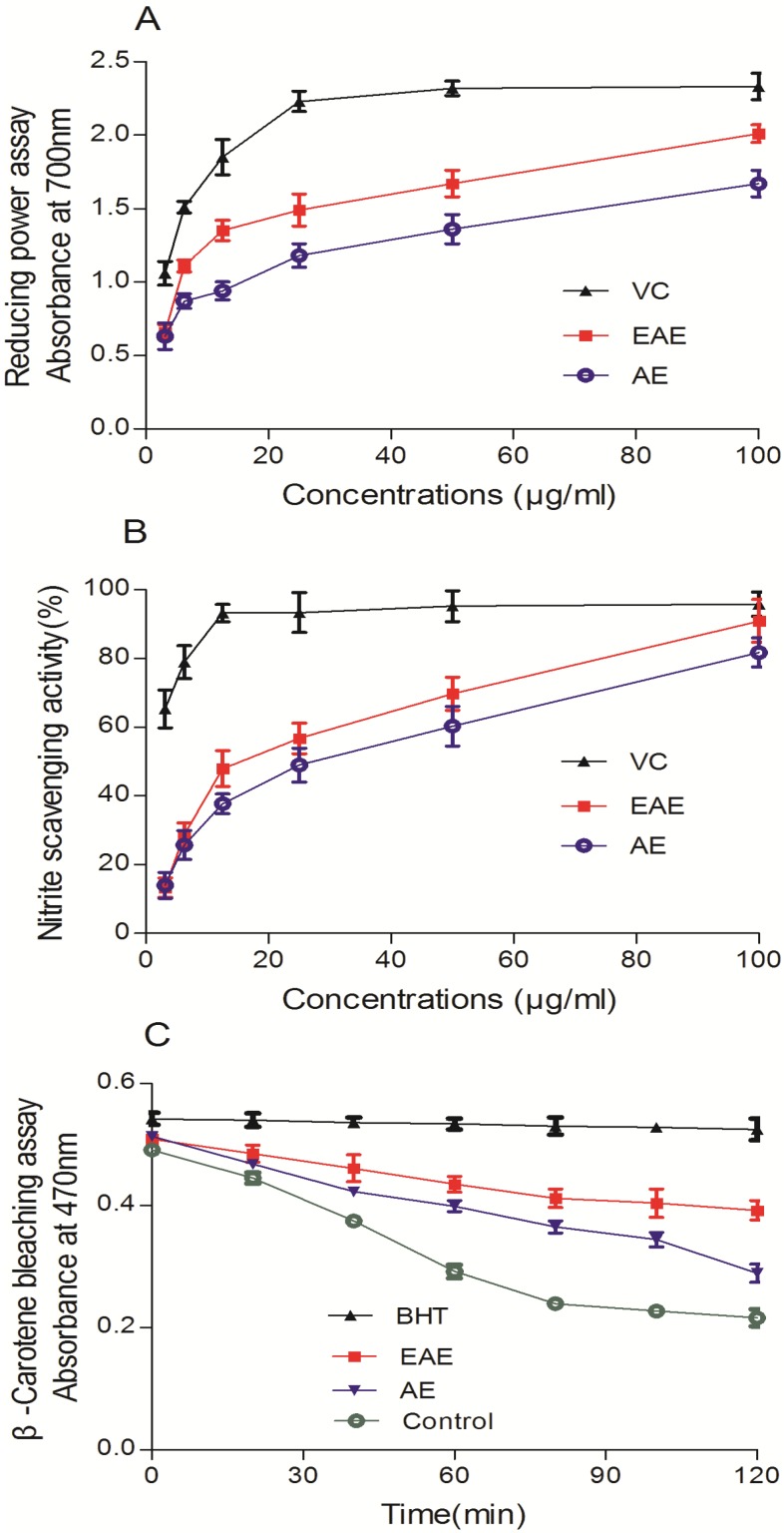
*In vitro* free radical scavenging of ethyl acetate and aqueous extracts from *V. ciliata* Fisch. (**A**) Ferric-reducing antioxidant power of VCFE and VC. (**B**) Nitrite scavenging activity of VCFE and VC. (**C**) β-Carotene bleaching assay of VCFE and BHT. Data are presented as means ± SD (*n* = 3). VCFE, *V.* *ciliata* Fisch. Extracts; VC, Vitamin C; EAE, ethyl acetate extracts of *V.* *ciliata* Fisch.; AE, aqueous extracts of *V.* *ciliata* Fisch.; BHT, 2, 6-ditert-butyl-4-methylphenol.

As shown in Figure 2A, the presence of VCFE as reductant caused the reduction of the Fe^3+^/K_3_Fe (CN)_6_ complex to Fe^2+^, and consequently, the Fe^2+^ could be monitored by measurement of the enhanced formation of Perl’s Prussian blue at 700 nm. In the broad range of 3.125–100 μg/mL, the phenomenon of concentration dependence was still obvious and ferric-reducing antioxidant power values (increased absorbance at 700 nm) of EAE and AE ranged from 0.66 to 2.01 and 0.63 to 1.67 respectively, while for VC it ranged from 1.06 to 2.33. Similarly, VCFE at the concentrations of 3.125–100 μg/mL also exhibited obvious scavenging activity against nitrite (Figure 2B). The scavenging activity of nitrite increased rapidly with the increase of sample concentration. At a concentration of 100 μg/mL, the inhibition ability was 90.9%.

The β-carotene bleaching assay is based on the radical adducts of carotenoid with free radicals from linoleic acid. The reductions in the absorbance of the β-carotene-linoleate emulsion in the presence of VCFE and the positive control, BHT, at a concentration of 25 μg/mL are shown in Figure 2C. All the samples and the standard inhibited bleaching of β-carotene in comparison with the negative control.

As shown in Table 1, at the concentration of 0.5 mg/mL, EAE and AE significantly slowed the reaction (*p* < 0.05), but their effects were weaker than those of the positive control, VC. However, the scavenging activity of EAE was more strong than that of AE (*p* < 0.05).

**Table 1:** Inhibition of pyrogallic acid auto-oxidation by *V.* *ciliata* Fisch. Extracts.

Sample	Control	EAE	AE	VC
*K_b_* value (×10^−2^)	10.41 ± 0.04	2.37 ± 0.12	6.74 ± 0.25	0.85 ± 0.02

EAE, ethyl acetate extract of *V.* *ciliata* Fisch.; AE, aqueous extract of *V.* *ciliata* Fisch.; VC, Vitamin C; Data are presented as means ± SD (*n* = 3).

### 2.3. CCl_4_-Induced Liver Damage Assays

#### 2.3.1. Effects of EAE on Serum Biochemical Parameter

**Figure 3:**
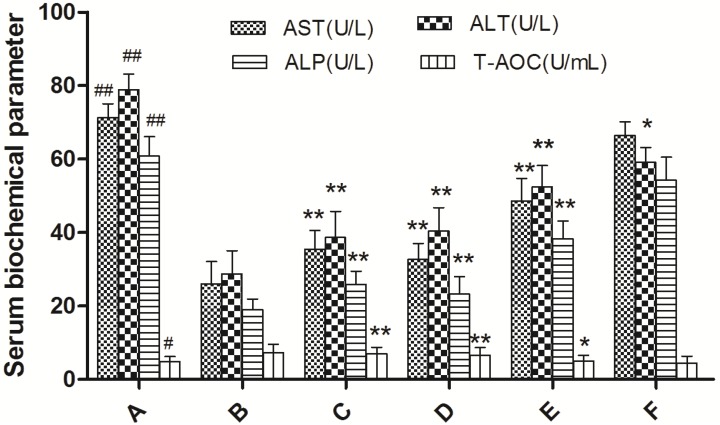
Effect of ethyl acetate extract of *V. ciliata* Fisch. (EAE) on the activities of serum ALT, AST, ALP, and T-AOC in CCl_4_-induced liver damage mice. Results are presented as the means ± SD (*n* = 10 animals in each group). (**A**) model control group; (**B**) normal control group; (**C**) groups administrated with bifendate at a dose of 150 mg/kg; (**D**) groups administrated with EAE at a dose of 600 mg/kg treatment group; (**E**) groups administrated with EAE at a dose of 300 mg/kg treatment group; and (**F**) groups administrated with EAE at a dose of 150 mg/kg treatment group. Notes: ^##^ *p* < 0.01, ^#^ *p* < 0.05, significant differences from the normal control group; ** *p* < 0.01, * *p* < 0.05, significant differences from the model control group.

We evaluated the hepatoprotective activity of EAE against CCl_4_-induced liver damage in mice by performing an analysis of serum ALT, AST, ALP and T-AOC. The serum biochemical parameter values are presented in Figure 3. The activities of the enzymes ALT, AST, ALP were significantly increased and T-AOC value was decreased in the CCl_4_ group compared to the control group (*p* < 0.001), reflecting the tissue damages in liver. Administration of EAE (150, 300 and 600 mg/kg) and bifendate had obviously reversed this elevation towards normal. This reverse effect of EAE was dose-dependent, and the high dose (600 mg/kg) created a therapy comparable to standard drug bifendate at 150 mg/kg.

#### 2.3.2. Effects of EAE on the MDA and GSH Levels as well as SOD Activities in Liver Homogenates

**Figure 4:**
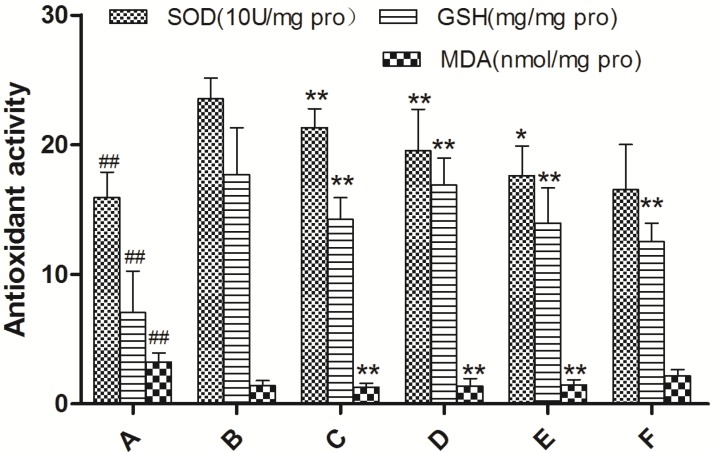
Effect of ethyl acetate extract of *V. ciliata* Fisch. (EAE) on SOD, CAT and GSH levels in liver tissue of CCl_4_-induced liver damage mice. Results are presented as the means ± SD (*n* = 10 animals in each group). (**A**) model control group; (**B**) normal control group; (**C**) groups administrated with bifendate at a dose of 150 mg/kg; (**D**) groups administrated with EAE at a dose of 600 mg/kg treatment group; (**E**) groups administrated with EAE at a dose of 300 mg/kg treatment group; and (**F**) groups administrated with EAE at a dose of 150 mg/kg treatment group. Notes: ^##^ *p* < 0.01, ^#^ *p* < 0.05, significant differences from the normal control group; ** *p* < 0.01, * *p* < 0.05, significant differences from the model control group.

The effects of different treatments on the SOD, MDA and GSH levels in the liver were shown in Figure 4. Compared to normal mice, CCl_4_ treatment significantly increased the MDA content and decreased the level/activity of GSH and SOD, suggesting stronger oxidative stress and lipid peroxidation in liver tissue (*p* < 0.001). Pretreatment with EAE (150, 300 and 600 mg/kg) and bifendate had more or less prevented this trend, according to the amount of VCFE (*p* < 0.05). When the dose reached 600 mg/kg, the results were as good as bifendate at 150 mg/kg.

#### 2.3.3. Histopathological Examination of Mice Liver

**Figure 5:**
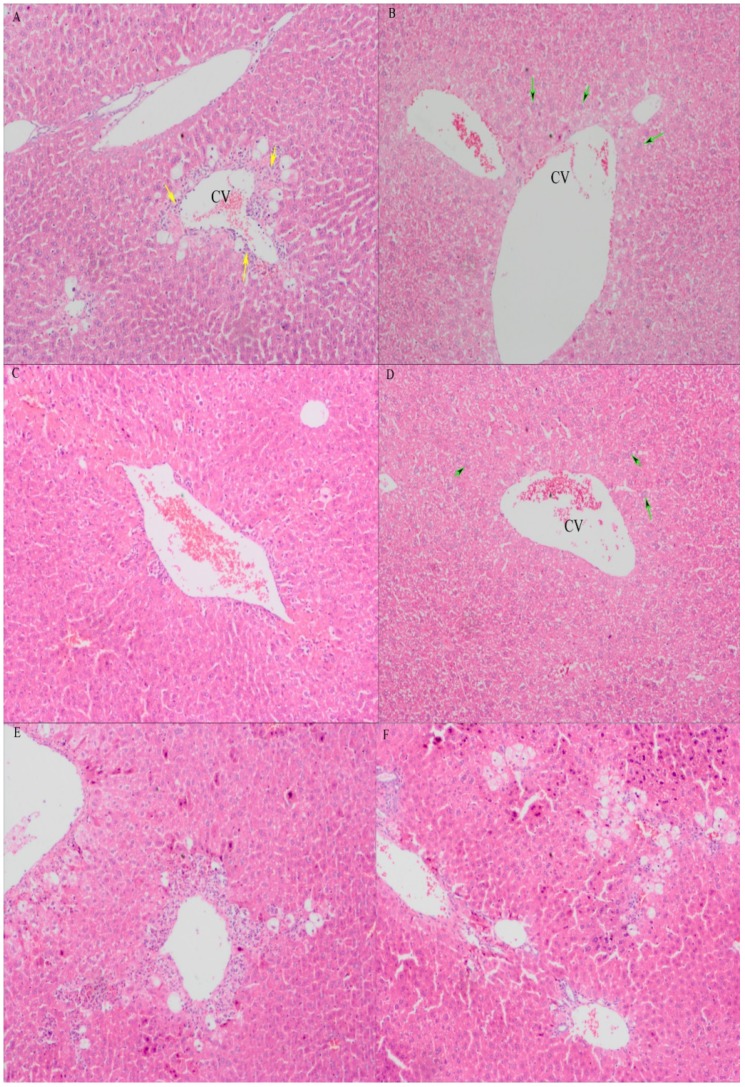
Preventive effects of ethyl acetate extract of *V. ciliata* Fisch. (EAE) against CCl_4_-induced liver histopathological changes in mice (original magnification of ×100). (**A**) model control group; (**B**) normal control group; (**C**) groups administrated with bifendate at a dose of 150 mg/kg; (**D**) groups administrated with EAE at a dose of 600 mg/kg treatment group; (**E**) groups administrated with EAE at a dose of 300 mg/kg treatment group; and (**F**) groups administrated with EAE at a dose of 150 mg/kg treatment group. The green arrows indicate normal cellular architecture with clear hepatic cell nucleus. The yellow arrows indicate the hepatic cell necrosis. CV: central vein.

In this study, histopathological observation of the liver was performed to further support the biochemical analysis evidence. The histology of the liver sections of the control group exhibited normal hepatic cells with well-preserved cytoplasm, prominent nucleus and nucleolus, visible central veins and thin sinusoids (Figure 5B). In contrast, the model group revealed the most severe damage of all the groups; the liver sections showed necrosis, massive fatty changes, broad infiltration of inflammatory cells, ballooning degeneration and the loss of cellular boundaries (Figure 5A). The maximum protection was observed at the dose of 600 mg/kg of EAE, and the liver sections of the mice from this group releaved minor pathomorphological changs that were almost comparable to the control and bifendate treated group. This histopathological observation was in good correlation with the biochemical results of serum hepatotoxic markers and other biochemical parameters (Figure 5C,D). However, histological changes in liver tissues from groups which treated at dose of 150, 300 mg/kg did not show significant protective effects against CCl_4_-induced hepatotoxicity (Figure 5E,F).

### 2.4. Discussion

Because phenolic and flavonoid compounds are recognized as the bases of the antioxidant activity of plant extracts, we determined the phenolic and flavonoid content of VCFE. It was found that the antioxidant activity of the ethyl acetate extract of *V. ciliata* Fisch. was superior to that of the aqueous extract (*p* < 0.05). This result was in line with the result that both the phenolic and flavonoid contents of EAE were more those of AE. Therefore, the ethyl acetate extract was chosen for evaluation of the hepatoprotective activity using hepatotoxicity induced by CCl_4_ in a mouse model and it was found to have good hepatoprotective effects evidenced by suppression of CCl_4_-induced oxidative stress in the livers of mice, and attenuating the morphological changes caused by CCl_4_. Moreover, the correlation between antioxidant and hepatoprotective activity was also investigated. The results suggested that the possible mechanism of this activity may be due to free radical-scavenging and antioxidant activity.

As it is known to all, CCl_4_ is the most extensively used chemical agent to investigate hepatoprotective activity in many exprimental animal models. CCl_4_ is biotransformed in the liver to the acive CCl_3_ radical, which reacts with oxygen to generate a trichloromethyl peroxyl radical (CCl_3_OO•), and then CCl_3_OO• reacts with lipids or proteins to cause lipid peroxidation along with other free radicals [11,12]. Therefore, free radical-scavenging is the most important way to protect the liver against hepatotoxicity induced by CCl_4_. In a previous study, five iridoid glycosides have been isolated from *V. ciliata* Fisch., and iridoid glucosides of the genus *Veronica* exhibited potent antioxidant activity [13,14]. In the present study, the results showed that EAE had strong scavenging activity on several radicals (DPPH, ABTS and superoxide radical). Reducing power assay, nitrite scavenging and β-carotene bleaching assay were also investigated, indicating that EAE was also a potent antioxidant.

In s previous study, it has been reported that administration of CCl_4_ in mice caused increases in ALT, AST and ALP activities, lipid peroxidation products, and a decrease in antioxidative enzymes [14]. In the present study, the increased serum level of ALT, AST and ALP enzymes in CCl_4_ treated mice confirmed the hepatic damage. Conversely, pretreatment with VCFE in different mice groups caused a significant decrease in serum level of ALT, AST, ALP enzymes and increase in T-AOC value of mice serum, indicating that this is involved in the maintenance of normal structure and function of cells, probably by redox and detoxification reactions. This finding contributes to the understanding that VCFE is one of the main active ingredients responsible for the hepatoprotective effect of *V. ciliata* Fisch.

It is reported that hepatic tissue damage induced by CCl_4_ caused lipid peroxidation and finally triggers production of MDA [15]. In agreement with the previous report about CCl_4_-induced oxidative stress [16], the present study showed that acute CCl_4_ treatment caused an increase of hepatic MDA concentration, and administration of EAE decreased the elevation of MDA levels, especially at the dose of 600 mg/kg. It is well-known that nonenzymatic antioxidant GSH can protect liver tissue against oxidative stress, and several endogenous antioxidant enzymes such as SOD can also convert reactive oxygen species (ROS) into less noxious compounds in living organisms [17]. In the present study, the activities of hepatic GSH and SOD in CCl_4_-treated mice were markedly weakened by administration of EAE, reflecting that EAE possesses potent hepatoprotective activity *in vivo*, which might contribute to its strong antioxidant activity. In agreement with the results of the biochemical parameters assay in serum and liver tissues, administration of VCFE reduced the histopathological alterations induced by CCl_4_. Histopathological examination also showed that the severe CCl_4_-induced pathological damage of mouse liver was markedly reduced by the administration of VCFE. This may be due to it preventing the toxic chemical CCl_4_ from forming highly reactive radicals. This suggests that the hepatoprotective effect of VCFE is a very encouraging finding, and the effect is mediated by antioxidative and free radical scavenging mechanisms.

## 3. Experimental

### 3.1. Chemicals

The diagnostic kits for AST, ALT, AKP, T-AOC, MDA, SOD, GSH and TP were purchased from Nanjing Jiancheng Bioengineering Institute (Nanjing, China). Linoleic acid, 2,2'-azino-bis(3-ethylbenzothiazoline-6-sulfonic acid) (ABTS), β-carotene, vitamin C (VC), 2,6-di-*tert*-butyl-4-methylphenol (BHT), and 2, 2-diphenyl-1-picrylhydrazyl (DPPH) were purchased from Sigma-Aldrich Chemical Co. (St. Louis, MO, USA). Bifendate (BDP) was purchased from Beijing Union Pharmaceutical Factory (Beijing, China). All other chemicals and reagents for the analysis in this study were of analytical grade.

### 3.2. Plant Material and Extraction

The herbs of *V. ciliata* Fisch*.* were bought from Tibet, China. A voucher specimen (No. 00721478) was identified by Dr. Jie Bai, School of Life Sciences, Sichuan University, and deposited in the Herbarium of Sichuan University. The locally collected herbs were shade dried and powdered. The powder (1 kg) was extracted at ambient temperature (22–25 °C) successively with petroleum ether (2000 mL × 3), ethyl acetate (2000 mL × 3). During extraction with solvents, the solvent was changed after every 24 h. The ethyl acetate from the pooled extracts was removed by distillation under reduced pressure at 40–45 °C to afford EAE (42.5 g). Moreover, the powder (500 g) was macerated with distilled water (5:l) at room temperature for 12 h, and then boiled for 1 h. After filtration, the extract was dried into the powder by a vacuum-drier at 60 °C to afford AE (12.6 g). 

### 3.3. Determination of Total Phenolic and Flavonoid Content

The total phenolic contents of AE and EAE were determined using the Folin–Ciocalteau reagent according to the previously described procedure [9]. The results were expressed in mg of gallic acid equivalents per g of dry extract. The total flavonoid contents were determined according to colorimetric assay [9], with rutin as standard. The results were expressed in mg of rutin equivalents per g of dry extract.

### 3.4. In vitro Antioxidant Activity of the Extracts

#### 3.4.1. DPPH Radical Scavenging Assay

The scavenging activity on DPPH radical was evaluated according to an improved DPPH assay [18] with slight modifications. Briefly, 2 mL of the extracts at different concentrations (3.25–100 μg/mL, dissolved in ethanol) were mixed with 2 mL of DPPH solution (0.1 mM, in ethanol). VC was used for comparative purpose. Then, the mixture was shaken evenly and allowed to stand at room temperature in the dark for 30 min before the absorbance was measured at 517 nm. The radical scavenging activity was calculated as follows: DPPH radical scavenging activity (%) = [1 − (Ai − As)/Ac] × 100, where Ac is the absorption of the negative control, Ai represents the absorption of the experiment group and As represents the absorption of the sample background. The concentration of samples reducing 50% of free radical DPPH (IC_50_) was determined by plotting the percentage of inhibition against the sample concentrations.

#### 3.4.2. ABTS radical Scavenging Assay

The ability of VCFE to scavenge the ABTS cation radical was determined by the method previously published with minor modifications [19]. The ABTS + solution was prepared by reacting 7 mM ABTS with 2.45 mM potassium persulphate (final concentrations, both dissolved in phosphate buffer, 0.2 M, pH 7.4) at room temperature in the dark for 12–16 h. This solution was then diluted with phosphate buffer to obtain an absorbance of 0.7 ± 0.02 at 734 nm. 100 μL of various concentrations (3.25–100 μg/mL, dissolved in ethanol) of the extracts were mixed with 100 μL of the diluted ABTS + solution. The reaction mixture was incubated at 30 °C for 30 min, and absorbance at 734 nm was recorded. The level of radical scavenging was calculated using the equation described above for DPPH. VC was used as a reference.

#### 3.4.3. Reducing Power Assay

The reducing power of VCFE was measured by previous method [20]. Briefly, 1.0 mL of different concentration VCFE solutions (3.25–100 μg/mL, dissolved in ethanol) was mixed with 2.5 mL of phosphate buffer saline (0.2 M, pH 6.6) and 2.5 mL of 1% (*w*/*v*) K_3_Fe(CN)_6_ solution. After incubation at 50 °C for 30 min, 2 mL of 10% trichloroacetic acid (TCA) was added. Then 2.0 mL of the upper layer was combined with 2.0 mL of distilled water and 1 mL of 0.1% (*w*/*v*) FeCl_3_ solution. The absorbance was analysis at 700 nm (BHT was used as a positive control). Increased absorbance of the reaction mixture indicates a greater reducing power.

#### 3.4.4. Superoxide Radical Scavenging Assay

The activity of VCFE to scavenge superoxide radicals was determined by a pyrogallic acid auto-oxidation system with minor modifications [21]. Briefly, 0.5 mL of sample or standard (0.5 mg/mL, dissolved in ethanol) mixed with 3.3 mL of Tris–HCl buffer (50 mM, pH 8.2) were incubated for 10 min at room temperature, then added 200 μL of pyrogallic acid (3 mmol/L, prepared in 10 mmol/L HCl). The absorbance of the reaction mixture at 320 nm was measured after 30 s and then every 10 s for 90 s. The auto-oxidation rate constant (*Kb*) of pyrogallic acid was calculated from the curve of A_320 nm_ *vs.* time. The control did not contain VCFE and a concentration of VC identical to the samples was used as a reference. The inhibitory actions of VCFE on the auto-oxidation rate of pyrogallic acid correlated with their ability to scavenge superoxide radicals.

#### 3.4.5. Nitrite Scavenging Assay

The activity of VCFE to scavenge nitrite was measured by previous method [21] with slight modifications. Briefly, 0.3 mL of NaNO_2_ (5 μg/mL) was blended with 1 mL of VCFE or standard at various concentrations (3.25–100 μg/mL, dissolved in ethanol), then 0.1 N HCl was added dropwise until the pH was up to 2.0. After the mixture incubated at 37 °C for 30 min, the reaction mixture was immediately mixed with 0.3 mL of sulfanilic acid (0.4%) and then incubated at room temperature for 5 min. Afterwards, 0.3 mL of *N*-ethylenediamine (0.2%) and 2.0 mL of ultrapure water were added to the above mixture, and the samples were incubated for another 15 min at 25 °C, followed by absorbance analysis at 538 nm. The level of nitrite scavenging was calculated using the equation described above for DPPH. VC was used as a reference.

3.4.6. β-Carotene Bleaching Assay

Antioxidant activity of VCFE was evaluated according to the β-carotene bleaching method [17]. Briefly, 3 mL of β-carotene solution in chloroform (0.2 mg/mL) was pipetted into a round-bottom flask, then 0.05 mL of linoleic acid and 0.5 mL of Tween 40 were added successively. After the chloroform was removed in a rotary evaporator at 30 °C, 200 mL of ultrapure water was added to wash out the residue. The reaction mixtures contained 5 mL of the above emulsion and 0.2 mL of various concentrations (3.25–100 μg/mL, dissolved in ethanol) of samples or the standard and were incubated at 50 °C in a water bath for 160 min. Absorbance readings were then recorded at every 20 min. Lipid peroxidation (LPO) inhibition was calculated using the following equation: LPO inhibition = [(As − Ai)/As] × 100 (As = absorbance of control of assay; Ai = absorbance 2 h later of assay). BHT was used as a reference.

### 3.5. Test Animals

Sixty (60) Kunming male mice (22.31 ± 1.25 g, about one month of age) were provided by Chengdu DOSSY Biotechnology Co., Ltd. (Chengdu, China). The mice were housed together under standard environmental conditions (22 ± 2 °C; 12 h light-12 h dark cycle), and allowed free access to tap water and a standard commercial pellet diet. They were allowed to acclimatize for 3 days before the experiments. All the experiments were performed in accordance with the Regulations of Experimental Animal Administration issued by the State Committee of Science and Technology of People’s Republic of China.

### 3.6. Hepatoprotective Experiments of the Extracts in CCl_4_-Injured Mice

The modeling method was performed according to a previous study [22] with slight modifications. Mice were randomly divided into six groups with 10 animals each. Group I (CCl_4_ treated model group) and group II (normal control) were given distilled water by gavage for 7 days consecutively. Group III received BDP (150 mg/kg) orally daily for a period of 1 week, which served as a positive control. Groups IV–VI were administered EAE orally (600, 300 and 150 mg/kg, representative of low, medium and high dosage, respectively) daily for 1 week. Two hours after the last dosing, the mice of groups II–VI were intraperitoneally injected with CCl_4_ (0.12%, *v*/*v*, dissolved in olive oil, 10 mL/kg body weight) to induce acute liver injury, while the mice in the normal group were administered with equal volume of olive oil.

After 2 h, all the groups were fasted strictly for 16 h, but allowed to drink water *ad libitum*. At the end of the experimental period, blood and livers were obtained immediately after the animals were sacrificed by cervical dislocation. Blood samples were collected from all animals from retro-orbital venous plexus for biochemical variables analysis. Liver samples were dissected out and washed immediately with ice cold saline to remove as much blood as possible, and immediately stored at −70 °C until analysis. An extra sample of liver was excised and fixed in 10% formalin solution for histopathologic analysis. Sections (5 μm thick) were cut and stained with hematoxylin and eosin (H&E) for the histological examination.

The blood samples were centrifuged (2500 rpm, 15 min, 4 °C for serum collection. Serum analysis of various liver marker enzymes such as ALT, AST, ALP and T-AOC were detected using commercial reagent kits obtained from the Institute of Biological Engineering of Nanjing Jiancheng (Nanjing, China) according to the instruction manuals. All the examinations were conducted in triplicate, and the average counts were acquired from each individual sample.

Liver tissues were homogenized with corresponding buffer according to the protocols of commercially available kits (Jiancheng Institute of Biotechnology, Nanjing, China) and centrifuged at 2500 rpm for 20 min at 4 °C. The supernatant was collected and used for the determination of protein and enzymatic studies as described below. Hepatic levels of MDA, GSH and SOD were regarded as unfailing indicator of antioxidant status of tissues [23]. Here, MDA in liver homogenate was assayed by the method of TBA, and GSH and SOD were assayed as described by the manufacturer’s instruction of commercially available diagnostic kits. The protein concentration in homogenates was also measured by the method of Coomassie brilliant blue [24]. The measurements were also performed in triplicates, and the average counts were acquired from each individual sample as well.

### 3.7. Histological Analysis of Liver

The liver samples were sectioned and stained with haematoxylin-eosin (H&E) and subsequently examined under a light microscope (Olympus, Tokyo, Japan) for general histopathology examination. The extent of hepatic damage was evaluated on H&E slides. Histopathological changes in the sections were observed by a light photomicroscope. Finally, the images were examined and evaluated for pathological change analysis.

### 3.8. Statistical Analysis

Statistical analysis was performed using SPSS 15.0 software (SPSS Inc., Chicago, IL, USA). Comparisons between multiple groups were evaluated using one-way analysis of variance (ANOVA) followed by LSD *t*-test. Data were expressed as mean ± SD and *p* < 0.05 was considered as statistically significant.

## 4. Conclusions

Based on the results from both *in vitro* and *in vivo* experiments, the present investigation demonstrated, for the first time, that EAE possesses strong antioxidant activities and significant protective effect against acute hepatotoxicity induced by CCl_4_, and this is supported by the evaluation of liver histopathology in mice. It indicated that antioxidant activities and protective effect against acute hepatotoxicity of *V. ciliata* Fisch. was concentrated in the EAE. The results suggested that the possible mechanism of this activity may be due to free radical-scavenging and antioxidant activity.
